# Peritoneal pseudomyxoma after incomplete appendectomy

**DOI:** 10.1515/pp-2020-0119

**Published:** 2020-08-20

**Authors:** Dahbia Djelil, Anthony Dohan, Marc Pocard

**Affiliations:** Department of Digestive and Oncologic surgery, Hôpital Lariboisière-AP-HP, Université Paris Diderot-Paris 7, Paris, France; Department of Radiology, Hôpital Cochin, AP-HP, Paris, France; Université de paris, CAP-PAris Tech, INSERM U1275, Lariboisière Hospital, Paris, France; Department of Digestive and Oncologic surgery, Hôpital Lariboisière-AP-HP, Université Paris Diderot-Paris 7, Paris, France

**Keywords:** peritoneal pseudomyxoma, pseudomyxoma peritonei, residual appendix

**Figure 1:**
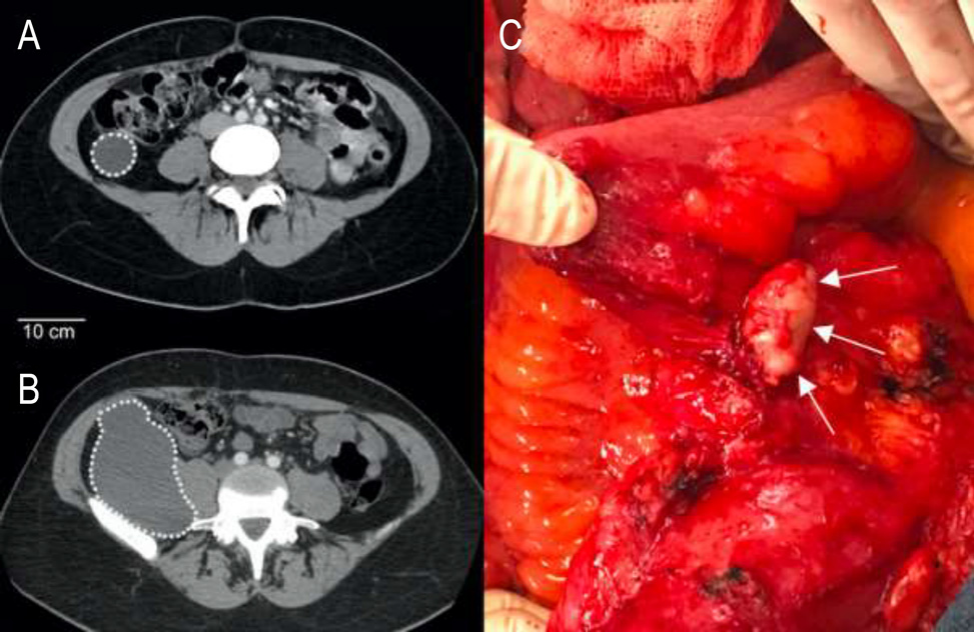
(A, B) Evolution of remnant tips of appendix generating a mucocele after 17 years and a pseudomyxoma peritonei (PMP) of low grade after 24 years. (C) Evolution of remnant tips of appendix generating a pseudomyxoma peritonei (PMP) of low grade. Per operative aspect with white arrow, on the last small bowel loop, of a remnant tips of appendix origin of the PMP.

A 41-year-old woman was referred because of abdominal pain, reporting on an appendectomy 24 years ago. At that time, she developed a postoperative intra-abdominal abscess and was told that the tip of the appendix had been left in the abdomen. Seventeen years later, a CT-scan showed a 6 cm cystic tumor at the caecum base (Figure 1A). The treatment was conservative. The abdominal CT-scan now showed a 15 cm large tumor (Figure 1B). A low-grade pseudomyxoma peritonei (PMP) with a Peritoneal Cancer Index (PCI) of 6/39 was diagnosed.

A 59-year-old man presented with abdominal pain nine years after a laparoscopic appendectomy. He reported on an intra-abdominal abscess eight months postoperatively, requiring radiological drainage. A low-grade PMP (PCI 12) was diagnosed, and an appendix tip remnant identified (Figure 1C).

A PMP can arise from an appendiceal tip remnant. A history of appendectomy does not exclude an appendiceal origin of PMP.

